# Dr. Edwin L. Wood

**Published:** 1912-07

**Authors:** 


Dr. Edwin L. Wood of the staff of the Jackson Sanitarium, Dansville, died in Beausoil, France, June 2, aged 51. He was a graduate of Buffalo, 1888. We regret the lack of definite data for a more extended notice of our classmate. We can only say that he was a diligent student, a conscientious practitioner, and a noble man.

